# Graphene Oxide-alginate Hydrogel for Drawing Water through an Osmotic Membrane

**DOI:** 10.1021/acsomega.2c03138

**Published:** 2022-10-18

**Authors:** Adetunji Alabi, Cyril Aubry, Linda Zou

**Affiliations:** † Department of Civil Infrastructure and Environmental Engineering, Khalifa University of Science and Technology, 127788Abu Dhabi, United Arab Emirates; ‡ Department of Research Laboratories Operations, Khalifa University of Science and Technology, 127788Abu Dhabi, United Arab Emirates

## Abstract

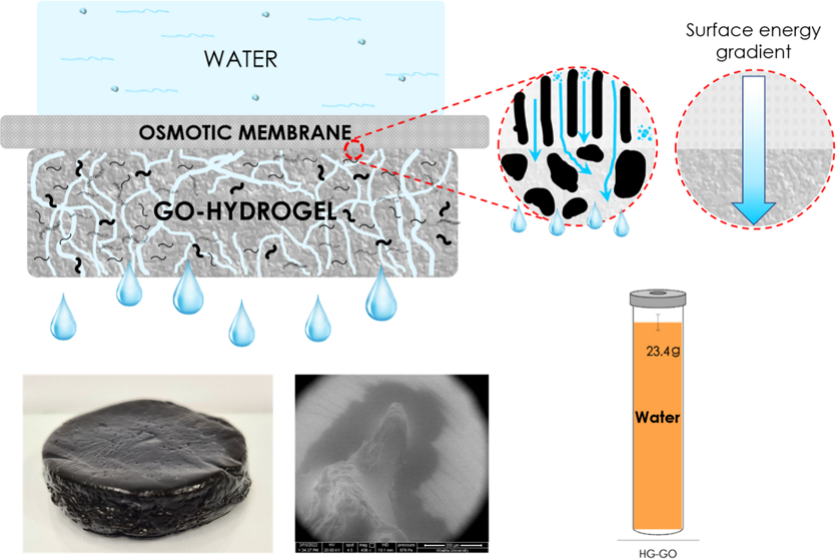

We report the preparation and evaluation of graphene oxide (GO)-enhanced alginate hydrogels for drawing water across an osmotic desalination membrane. GO-incorporated calcium alginate hydrogels (GO-HG) and pure calcium alginate hydrogels (P-HG) were synthesized for this study. Environmental scanning electron microscopy, water contact angle, and water uptake tests showed both samples to be strongly hydrophilic. The synthesized hydrogels demonstrated the ability to successfully and continuously draw water through a selective osmotic membrane in experiments. This was driven by the surface energy gradient-induced negative pressure between the more hydrophilic hydrogel and less hydrophilic membrane surface. The GO-HG was found to draw 21.2% more water than the P-HG, owing to the flexible GO nanosheets, which can be easily incorporated into the hydrogel framework. The GO nanosheets not only offer more hydrophilic functional sites but also enhance the connectivity within the alginate hydrogel framework so as to enhance the water production performance. The average amount of water drawn through the membrane by the GO-HG and the P-HG is 23.4 ± 0.9 g and 19.3 ± 1.8 g, respectively. It was found that no external stimuli were needed as water flows through the hydrogel due to gravitational force. The GO-enhanced alginate hydrogel, combined with the osmotic membrane, is a promising surface energy gradient-driven functional material for water purification and desalination without applying external pressure.

## Introduction

Hydrogels are synthetic or natural materials made up of polymer chains that are cross-linked by either physical or chemical bonds and are able to entrap large volumes of water courtesy of the high concentration of hydrophilic groups present in their polymer chains.^1^ An important characteristic of hydrogels is their ability to go through reversible volume change in response to changes in external stimuli. Some of the stimuli that have been used to produce desired changes in hydrogel systems are temperature,^2^ electric fields,^3^ hydrostatic pressure,^4^ pH,^5^ light,^6^ and solution concentration.^7^ Hydrogels have been successfully used in numerous applications such as tissue engineering and regenerative medicine,^8,9^ drug delivery,^10^ biosensors,^11^ food,^12^ agriculture,^13−15^ water treatment,^16,17^ and energy applications^18−20^ Among the hydrogels obtained from natural materials, alginate hydrogels are one of the most promising types because of the abundance of sources of alginate in nature.

Alginate is a natural, non-toxic, biodegradable, and biocompatible linear polysaccharide that is sourced primarily from brown algae.^21^ It is also sometimes obtained from certain kinds of bacteria.^22,23^ It is a copolymer made up of residues of β-d-mannuronic acid (M) and α-l-guluronic acid (G) groups.^24,25^ The acid groups are arranged in homopolymeric regions of M–M blocks and G–G blocks and in heteropolymeric regions of the M–G blocks.^26−28^

Alginate hydrogels are formed when polyvalent cations take part in the interchain ionic binding between the G blocks in the alginate polymer chain. The polyvalent cations act as cross-linkers that stabilize the alginate chains, thereby forming a three-dimensional polymeric network that has cross-linked chains combined with hydrophilic flexible chains.^24^ The hydrophilicity,^29^ biocompatibility,^30,31^ low toxicity,^32−34^ mild gelation conditions,^35^ ease of handling, and low cost^36−38^ of alginate hydrogels make them suitable for many applications. In the food industry, alginate hydrogels are typically used as gelling and encapsulation agents.^39^ In wastewater treatment, alginate hydrogels are used for the removal of dyes and heavy metals.^40,41^ In the biomedical field, alginate hydrogels are used for drug delivery,^42,43^ wound dressings,^44^ enzyme immobilization,^45^ encapsulating and releasing viral vectors in gene therapy,^46^ and bone tissue engineering.^47^ Research also shows that the incorporation of nanomaterials in alginate hydrogels can lead to improvements in the properties and performance of the hydrogels.^28,40,48,49^

Alginate hydrogels are capable of absorbing and retaining large amounts of water. Despite the hydrophilicity of alginate hydrogels, their potential to produce freshwater by attracting water molecules across semipermeable osmotic membranes has not been investigated and reported. However, the water-drawing capacity of alginate is limited by the fixed number and density of hydrophilic chains within its cross-linked polymeric network. There is a trade-off between increasing the mass percentage of alginate precursor in the synthesis and the mechanical strength and stability of the cross-linked polymer. Other methods of enhancing the water-drawing capability without compromising its physical strength are needed.

Graphene oxide (GO) is a 2D nanomaterial synthesized by a chemical method such as Hummers method^50^ via the oxidation of graphite flakes. The synthesized GO nanosheets have many polar oxygen functional groups to render the nanosheets hydrophilic and well dispersible within an aqueous medium.^51^ GO’s flexible physical properties and chemical tenability make them a good candidate to be incorporated in polymers. It is anticipated that flexible GO nanosheets can be incorporated nicely and offer more hydrophilic functional sites and enhance the connectivity within the alginate hydrogel framework, so as to enhance the water production performance of GO-alginate hydrogel framework.

Inspired by the water-drawing agent concept^52^ in osmotic desalination membrane processes, such as forward osmosis (FO), a suitable water-drawing agent for such applications should meet the following criteria: first, the water-drawing agent should have a relatively high osmotic pressure; second, the diluted water-drawing agent should be able to be easily and economically reconcentrated and/or recovered; and third, the water-drawing agent should exhibit minimized internal concentration polarization in the FO process.^53^ Although the common water-drawing agent in FO is in liquid form, for example, saline solution, in recent years, other non-liquid form of water-drawing agents have been reported, such as functionalized magnetic nanoparticles, thermoresponsive polyelectrolyte solutions, and stimuli-responsive polymer hydrogels.^52^ The polymer hydrogels’ swollen volume is reversible in response to external environmental stimuli, including temperature, light, pressure, solvent composition, and pH.^54^ Until now, several issues still need to be addressed, including the difficulties in regeneration and in continuous operation.^55−57^

A well-designed hydrogel can produce high-swelling pressure to draw water across the osmotic membrane and can also be regenerated by external stimuli, such as temperature and pH. Moreover, they have the advantage of low reverse ion diffusion rates because hydrogels are insoluble in water. Hydrogels made from crosslinked poly(sodium acrylate) (PSA) (or other polyelectrolytes)^58^ demonstrated good water flux in FO because of the strong hydration and ionization interactions between PSA and water molecules to reduce the water chemical potential and to increase the chemical potential gradient across the membrane. However, water recovery from PSA hydrogel is not effective even with simultaneous heating and squeezing by hydraulic pressure–only a small portion of water is recovered. Hydrogels face the obstacle of having a low water flux compared to conventional water-drawing solutes in solution. Therefore, hydrogels made from new materials need to be thoroughly investigated in order to identify their niche applications in the FO process for water production.

We have yet to come across any research work in which alginate hydrogels were used to draw water through an osmotic membrane. In the research works we encountered, other types of hydrogels other than alginate were used.^54,56,59−63^ Furthermore, in the research studies we have encountered, an additional dewatering step (which incurs additional time and costs) was required to recover the water drawn by the hydrogels. There appears to be a knowledge gap in understanding the feasibility and capacity of using alginate hydrogels to draw water by contacting an osmotic membrane: will the alginate hydrogel draw water across an osmotic membrane? Is a stimulus needed to desorb the water from the hydrogel? Will hierarchical 2D nanomaterials, such as GO nanosheets, incorporated in the hydrogel enhance the water production?

**Figure 1:**
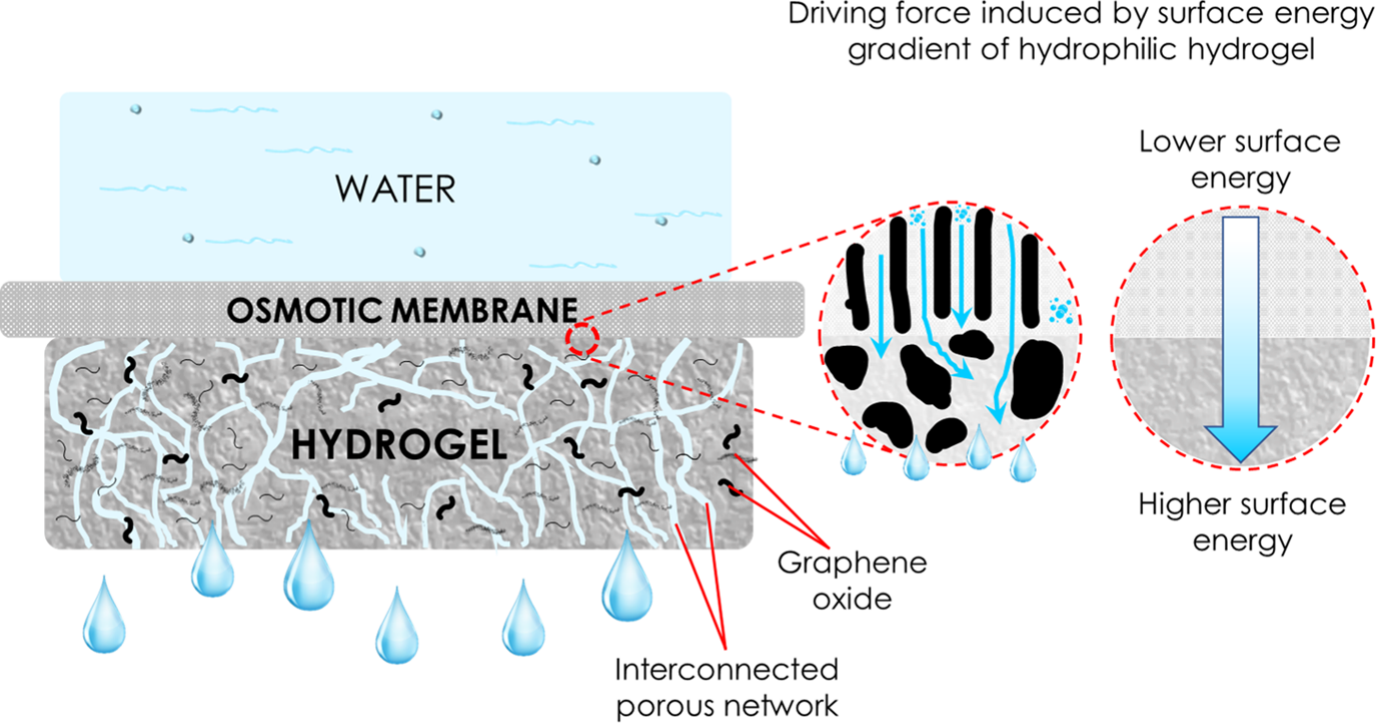
Concept schematic.

In this work, we report the synthesis of alginate-GO hydrogels and assess the potential of the hydrogels for water production through an osmotic membrane (Figure 1). The role of GO nanosheets in enhancing the alginate hydrogel property was also explored. Two types of calcium alginate hydrogels were produced; pristine calcium alginate hydrogels (P-HG) and GO-incorporated hydrogels (GO-HG) were synthesized, characterized, and used in bench-scale water production feasibility tests. These hydrogels are eco-friendly and nature-inspired. Furthermore, they are reusable without the need for a recovery/regeneration step.

## Materials and Methods

### Materials

Calcium chloride (CaCl_2_), sodium alginate (Na alginate), potassium permanganate (KMnO_4_), sodium nitrate (NaNO_3_), hydrogen peroxide (H_2_O_2_), hydrochloric acid (HCl), and sulfuric acid (H_2_SO_4_) were bought from Sigma Aldrich. Graphite flakes were bought from Bay Carbon Inc. (USA). Flat sheet CTA osmotic FO membrane was purchased from Sterlitech (USA). Deionized water (DI H_2_O) was used for all the experiments.

### Synthesis of Pure Hydrogel

**Figure 2:**
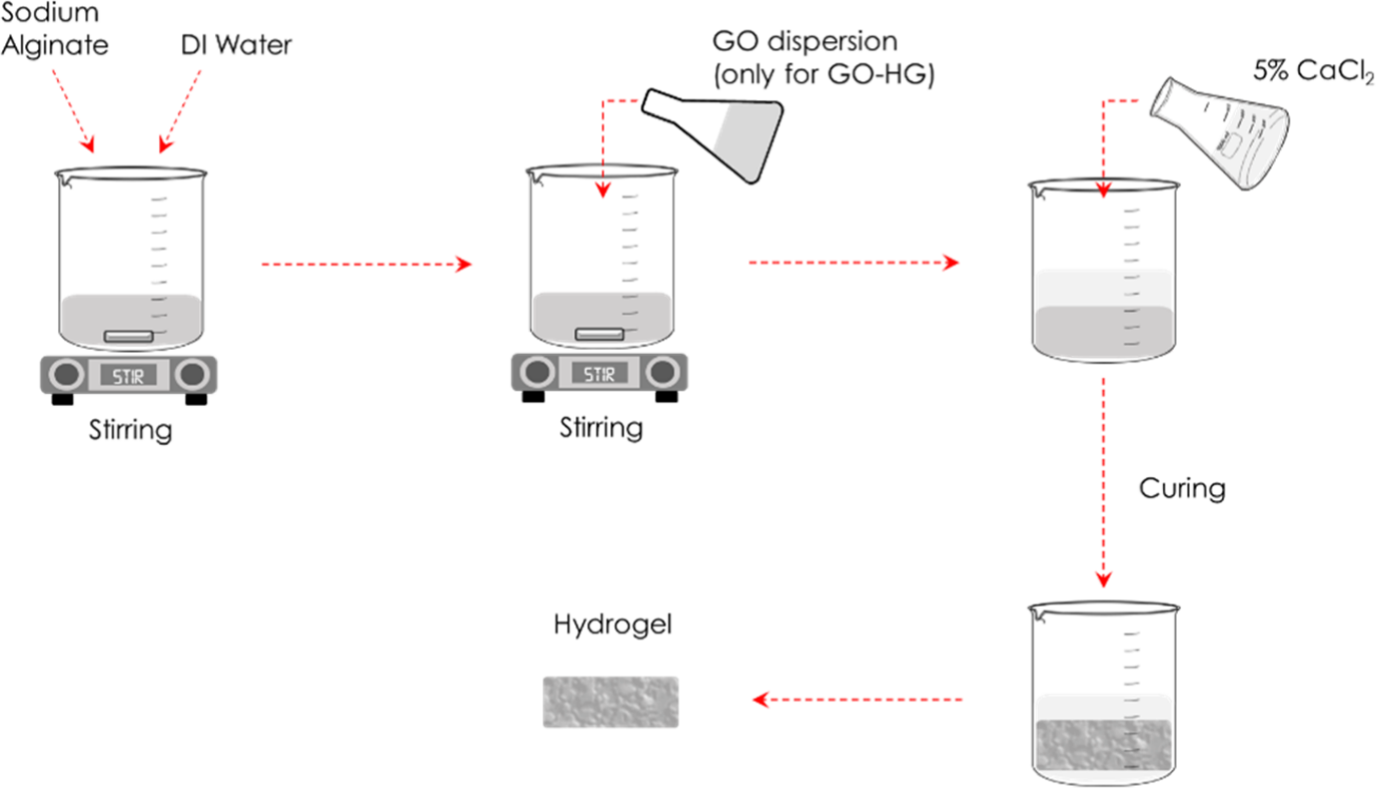
Synthesis of hydrogel samples.

**Figure 3:**
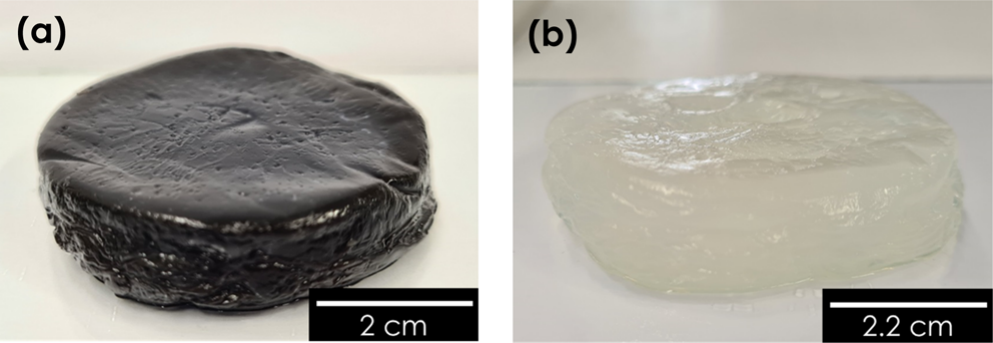
(a) GO-HG and (b) P-HG hydrogel samples.

Figure 2 shows a schematic of the hydrogel preparation procedure. Sodium alginate solution (1% w/v) was prepared by dissolving 10 g of sodium alginate powder in 990 mL of DI H_2_O. Then, 100 mL of the sodium alginate solution was transferred to a beaker. 40 mL of CaCl_2_ (5% w/v) was poured into 100 mL of the sodium alginate solution (1% w/v), and then the alginate was left to cure (i.e., toughen by cross-linking of the polymer chains) at room temperature for 20 h. The resultant hydrogel was then recovered and rinsed with DI H_2_O. A round cookie cutter (5 cm diameter) was used to cut out a piece of the pure hydrogel (Figure 3b) for water production trials. This was to ensure that the hydrogels used for the water production trials had a uniform surface area (ca. 19.63 cm^2^). The formulations of the starting and curing solutions are shown in Table 1.

**Table 1:** Composition of P-HG and GO-HG Hydrogels

	starting solution			curing solution
hydrogel/component	sodium alginate (g)	DI H_2_O (mL)	GO (g)	5% CaCl_2_ (mL)
pure HG	1	99	0	100
GO-HG	1	99	0.25	100

### Synthesis of GO

GO was prepared using a modified Hummers method.^50^ Briefly, 250 mL of H_2_SO_4_ was cooled to about 4 °C in an ice bath. This was followed by the addition of 2 g of graphite flakes and 1 g of NaNO_3_ to the cooled H_2_SO_4_. Then, the solution was stirred to achieve uniform mixing. 12 g of KMnO_4_ was slowly added to the solution. Thereafter, the solution was continuously stirred for 45 min in the ice bath. The solution was transferred to a water bath and stirred continuously for 2 h at 35 °C. Afterward, the solution was placed in an ice bath with continuous stirring applied. Then, 250 mL of DI H_2_O was slowly added to the solution. The solution was removed from the ice bath and stirred at room temperature for 2 h. Then, 500 mL of DI H_2_O was poured at once into the solution. This was followed by the dropwise addition of H_2_O_2_ till the solution turned golden yellow in color. The solution was then filtered in a vacuum filtration setup. The recovered graphite oxide cake was washed with 400 mL of HCl solution (1:10 vol %) and then vacuum filtered (this step was performed twice). The mud was then washed with DI H_2_O till the pH rose to ca. 6.5. Finally, the graphite oxide was diluted with DI H_2_O and then exfoliated with a probe ultrasonicator to produce GO nanosheets.

### Synthesis of GO Hydrogel

GO dispersion (10 g/L) was mixed with sodium alginate solution till a uniform solution was obtained. Then, 40 mL of CaCl_2_ (5% w/v) was poured into 100 mL of the resultant GO-sodium alginate solution and left to cure for 20 h. Just as with the pure hydrogel, the formed GO hydrogel was cut with a round cookie cutter (5 cm diameter). This cut GO-HG piece (surface area ca. 19.63 cm^2^) was used in the water production trials. The formulations of the starting and curing solutions are shown in Table 1. The produced GO-HG hydrogel is shown in Figure 3a.

### Characterizations

Fourier-transform infrared (FTIR) spectroscopy characterizations were performed with the attenuated total reflectance accessory of a Bruker Vertex 80v FTIR spectrometer in absorbance mode. The hydrogel samples were dried in air prior to the FTIR characterizations.

Raman spectroscopy characterizations were done with a WITec alpha300 RAS. The excitation wavelength used for the characterizations was 532 nm. Samples were prepared by applying drops of the sodium alginate solution (with and without GO) on glass slides and curing the solutions in 5% CaCl_2_ to produce hydrogel films. The prepared samples were then rinsed with DI H_2_O and dried in the air.

Scanning electron microscopy (SEM) micrographs were obtained using a FEI Nova NanoSEM 650. The hydrogel samples were coated with gold/palladium before the microscopy characterizations. Qualitative hydrophilicity tests were conducted using FEI Quanta 250 SEM in environmental mode. The humidity and the temperature were maintained at 100% and 5 °C, respectively, throughout the tests.

Water contact angle measurements were obtained using a Kyowa DropMaster water contact angle goniometer (model DM-501). The software for analysis was FAMAS. Samples for the characterizations were prepared on glass slides. Six measurements were taken for each hydrogel sample, and the average was used as the final value.

Topography characterizations of the hydrogels were assessed using an Asylum Research Cypher atomic force microscope in tapping mode. Samples were prepared as films on glass slides. The frequency of the tip was ∼150 kHz, and a scan size of 5 μm was used for all characterizations.

**Figure 4:**
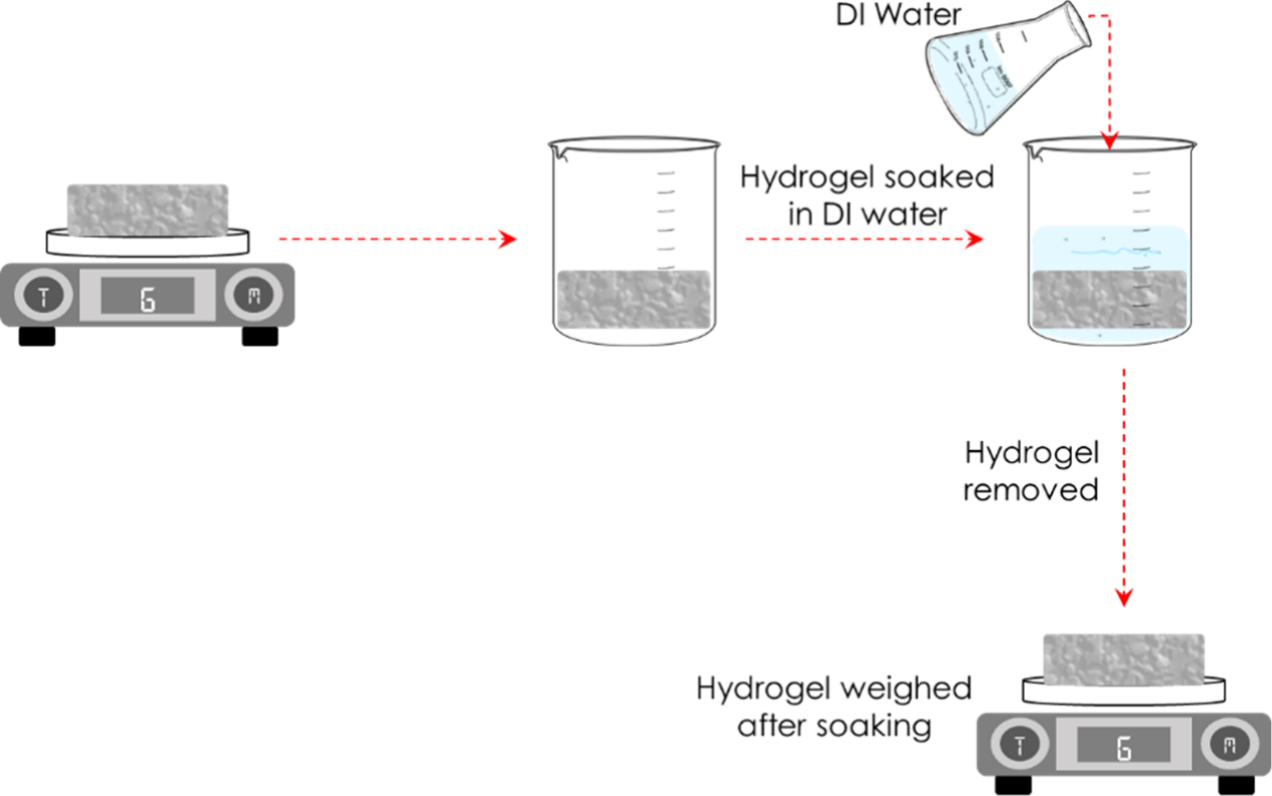
Diagrammatic illustration of water uptake tests.

Water uptake tests were carried out by first weighing the hydrogel and then immersing the hydrogel in 500 mL of DI H_2_O for 24 h, followed by measuring the weight of the hydrogels (see Figure 4). The water uptake was calculated according to eq 1
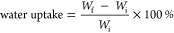
1where *W*_f_ is the weight of the hydrogel after soaking, and *W*_i_ is the weight of the hydrogel before soaking. This cycle was repeated for a total of 22 days, and the cumulative water uptake for each hydrogel sample was calculated at the end of the tests.

### Water Production Tests

**Figure 5:**
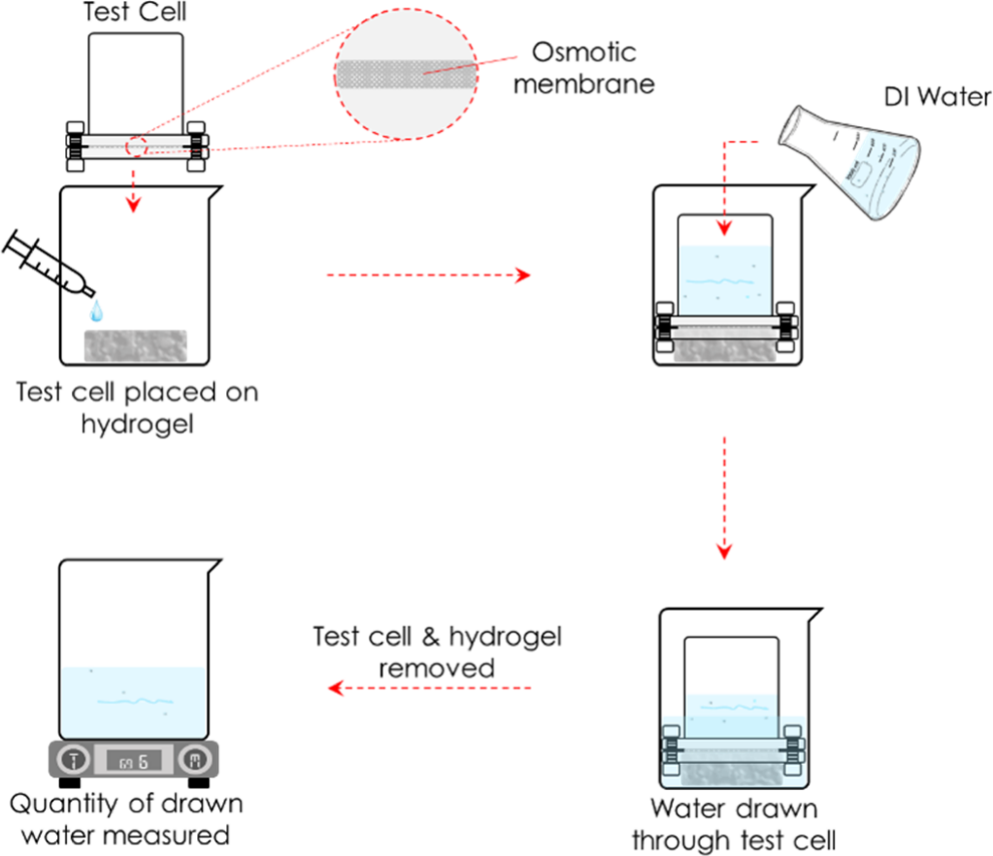
Schematic of water production tests.

**Figure 6:**
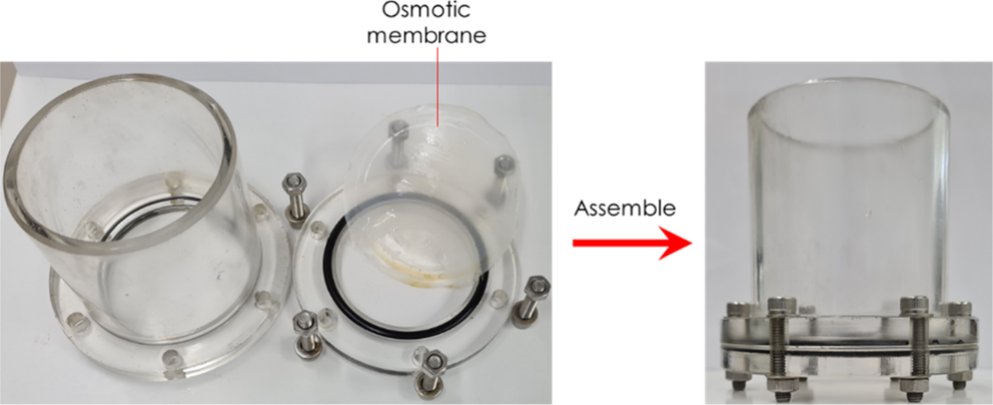
Test cell components and assembly.

An illustration of the water production test setup is shown in Figure 5. First, a piece of hydrogel sample was placed in an empty beaker; second, a piece of the osmotic membrane was assembled in the test cell, as shown in Figure 6; third, the assembled test cell was placed on top of the hydrogel to ensure close contact between the hydrogel and the osmotic membrane; finally, the test cell was filled with 300 mL of DI H_2_O. The setup was left undisturbed for 20 h, after which the quantity of free water in the beaker drawn by the hydrogel was measured. The water production test was repeated 2 more times for each hydrogel sample. Before each test, 3 mL of CaCl_2_ was poured on the hydrogel samples. CaCl_2_ solution was added to induce a concentration gradient, which initiated water transport from the DI H_2_O side of the membrane to the hydrogel side of the membrane. By adding CaCl_2_, the water chemical potential on the surface of the hydrogel was reduced, thus initiating the transport of water through the membrane and to the interconnected water channels of the hydrogel.

## Results and Discussion

### Surface Morphology

**Figure 7:**
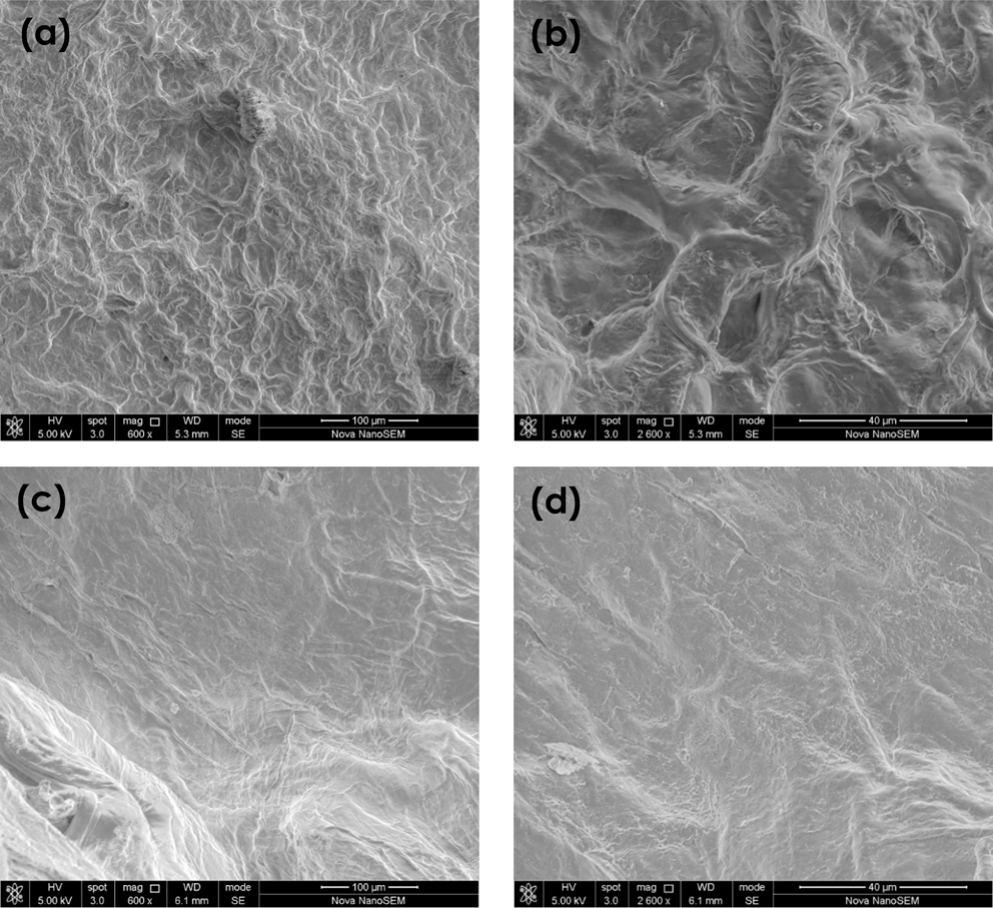
SEM images for GO-HG (a,b) and P-HG (c,d).

Figure 7a,b shows the morphology of GO-HG, and Figure 7c,d shows the morphology of P-HG. The surface of the GO-HG hydrogel appears to have a rougher morphology than that of the P-HG hydrogel owing to the presence of the additional 2D material (i.e., GO nanosheets) in GO-HG. A 20 wt % GO content resulted in significant differences in physical appearance and surface morphology (Figure 3). The GO-HG sample displayed a blackish color compared to the translucent pale color of the P-HG sample.

**Figure 8:**
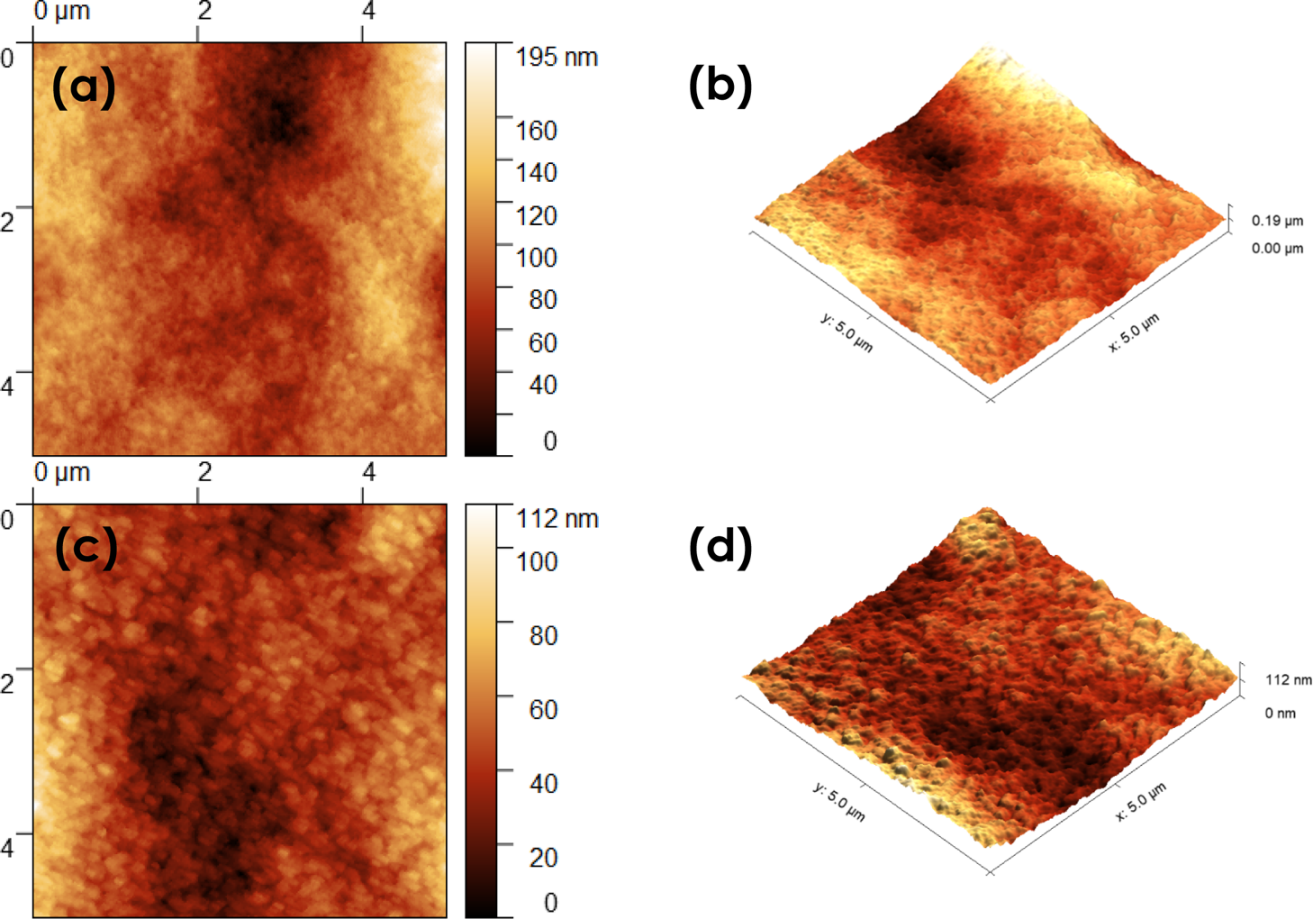
AFM images for GO-HG hydrogel (a,b) and P-HG hydrogel (c,d).

The surface roughness of the hydrogels was assessed from AFM topography experiments (Figure 8). The average surface roughness of the GO-HG hydrogel was 29.69 nm (SD = 5.96 nm) while that of the P-HG hydrogel was 14.28 nm (SD = 2.58 nm). This suggests that the GO-HG hydrogel had a rougher surface than the P-HG hydrogel. These results are consistent with the surface morphology observations of the SEM images.

### Surface Interactions with Water

**Figure 9:**
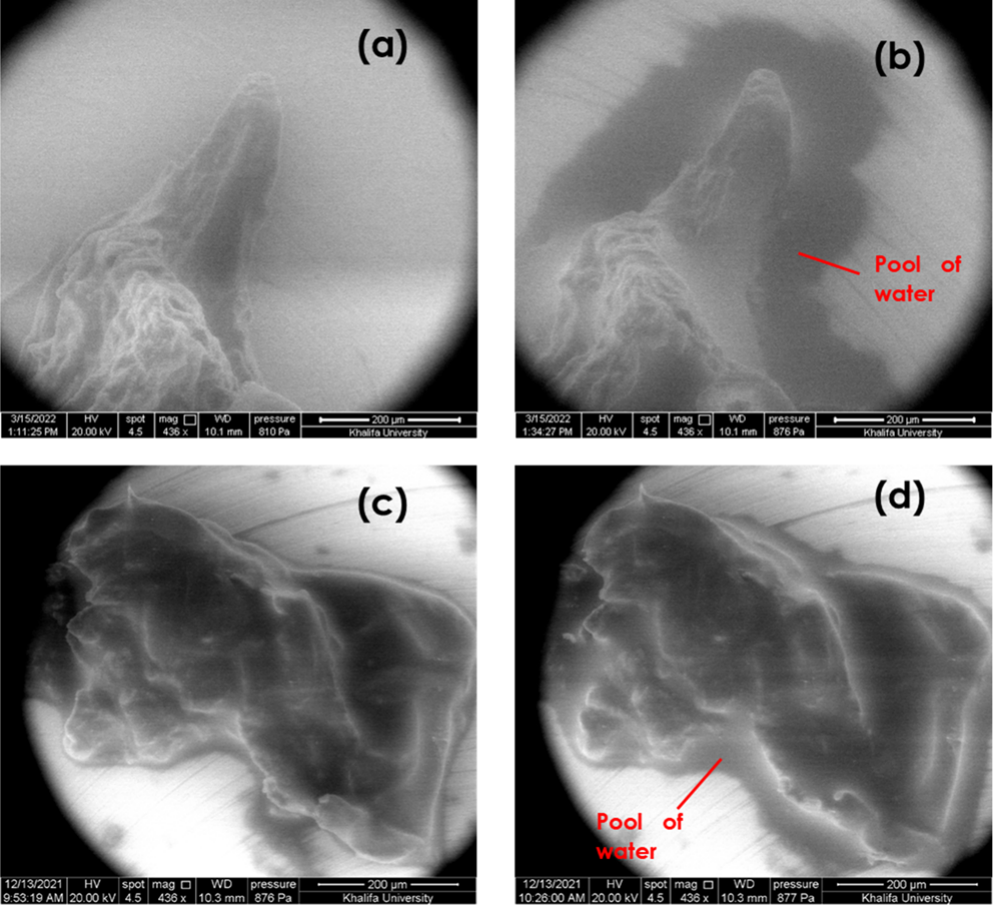
Micrographs from E-SEM tests showing the hydrogel’s affinity for water at the microscale. The qualitative wettability of the hydrogels is shown by the accumulated water which formed shadows around the perimeter of the hydrogel samples. (a) Dry GO-HG before the test, (b) wet GO-HG during the test, (c) dry P-HG before the test, and (d) wet P-HG during the test.

Surface interaction between the hydrogels and water vapor was observed using the environmental mode of a SEM. The relative humidity and the temperature were maintained at 100% RH and 5 °C, respectively, throughout the tests. Images from the environmental SEM (E-SEM) tests are shown in Figure 9. The micrographs show the hydrogel samples just before the tests were conducted (Figure 9a,c) and the hydrogel samples at the end of the 25 min tests (Figure 9b,d). From the images, it can be observed that after about 25 min, both hydrogel samples (GO-HG and P-HG) had a pool of water gathered around them, where the GO-HG showed a larger water pool relative to its sample size, whereas this gathered water is noticeably absent from the hydrogel samples at the beginning of the tests. These results serve as a qualitative demonstration of both hydrogels’ affinity for water vapor; they attracted the water vapor inside the E-SEM chamber and then the water vapor condensed to liquid water, shown as dark shadows in the images.

The results from the water contact angle characterizations are shown in Figure S1. The mean water contact angle of the P-HG was 16.1 ± 2.0° while that of the GO-HG was 24.2 ± 1.5°. The obtained results demonstrate the high hydrophilicity of both hydrogels. Furthermore, the lower water contact angle for the P-HG suggests that its surface is more hydrophilic than the surface of the GO-HG. Water uptake results (Figure S2) show that the P-HG absorbed more water than the GO-HG within the same duration. Over a period of 22 days, the cumulative water uptake for the P-HG was 59% whereas that for the GO-HG was 32%. These results suggest that the P-HG had a higher capacity to contain water than the GO-HG. This may be as a result of the higher hydrophilic nature of the P-HG, resulting in a greater capacity for the P-HG to attract and absorb water. For a hydrophilic material, although an increase in surface roughness increases the hydrophilicity of the material’s surface, changes in the surface energy of the material can also affect the wettability of the material’s surface.^64,65^ For the GO-HG, the incorporation of GO increased the surface roughness of the hydrogel; however, a slight increase in the water contact angle was observed. Since an increase in the surface roughness was not accompanied by an increase in wettability, the lower wettability can therefore be attributed to changes in the surface chemistry, whose effects superseded those of the surface roughness and thus resulted in a net decrease in the surface hydrophilicity. Although GO nanosheets have polar hydrophilic functional groups on their oxidized edges and planes, there are still local areas of GO nanosheets that are uncharged, and the 2D sheet structure could also cover up some hydrophilic groups on the alginate polymer chains and result in an overall slightly less hydrophilic GO-HG.

### Chemical and Structural Characterizations

**Figure 10:**
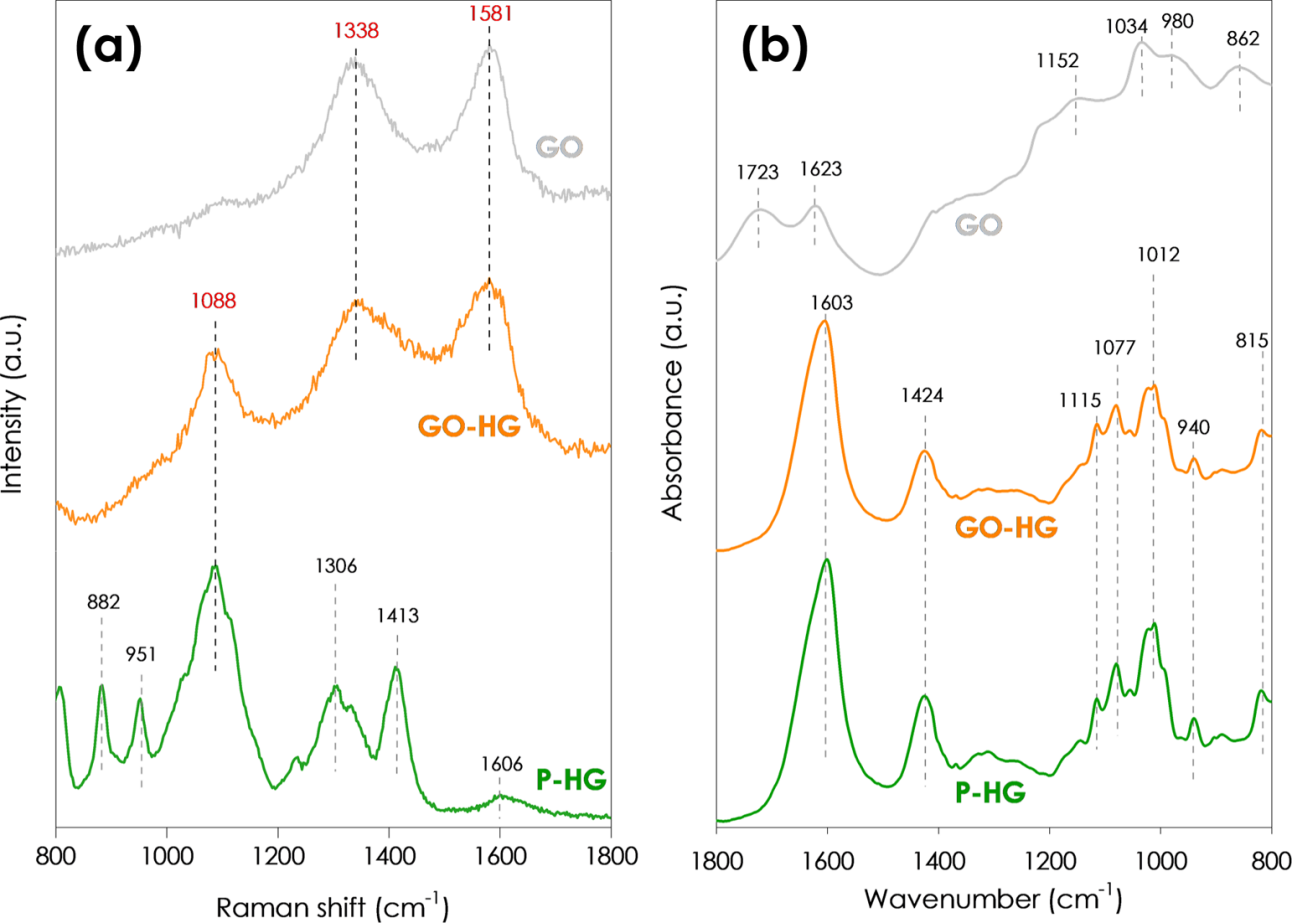
(a) Raman and (b) FTIR spectra for the GO, GO-HG, and P-HG samples.

Figure 10a shows the Raman spectra of the samples. Typical D and G bands of GO are present at 1338 and 1581 cm^–1^, respectively.^66−68^ The characteristic bands for calcium alginate^25,49,69^ are shown in the spectrum of the P-HG hydrogel. It can be observed that in the spectrum for the GO-HG, the bands at 882, 951, and 1606 cm^–1^ are not present. Furthermore, the bands at 345, 551, and 1088 cm^–1^ are present in both the GO-HG and the P-HG spectra. Also, bands at 1338 and 1581 cm, which correspond to the D and G bands of GO, are present in the spectrum for GO-HG but absent from that of P-HG. This confirms the successful incorporation of GO into the GO-HG hydrogel.

The FTIR spectra of the samples are displayed in Figure 10b. The spectrum for GO shows characteristic C=O stretch, C–O–C asymmetric stretch, and epoxide stretch vibrations.^70−72^ The spectra for both the P-HG and the GO-HG are similar and they both contain characteristic peaks for calcium alginate.^26,73,74^ Since there is no shift in the peaks of the P-HG and the GO-HG, we can surmise that no new functional groups were formed in the GO-HG and the integration of GO into the hydrogel was achieved via physical means.

### Evaluation of Water Production by GO-HG and P-HG

**Figure 11:**
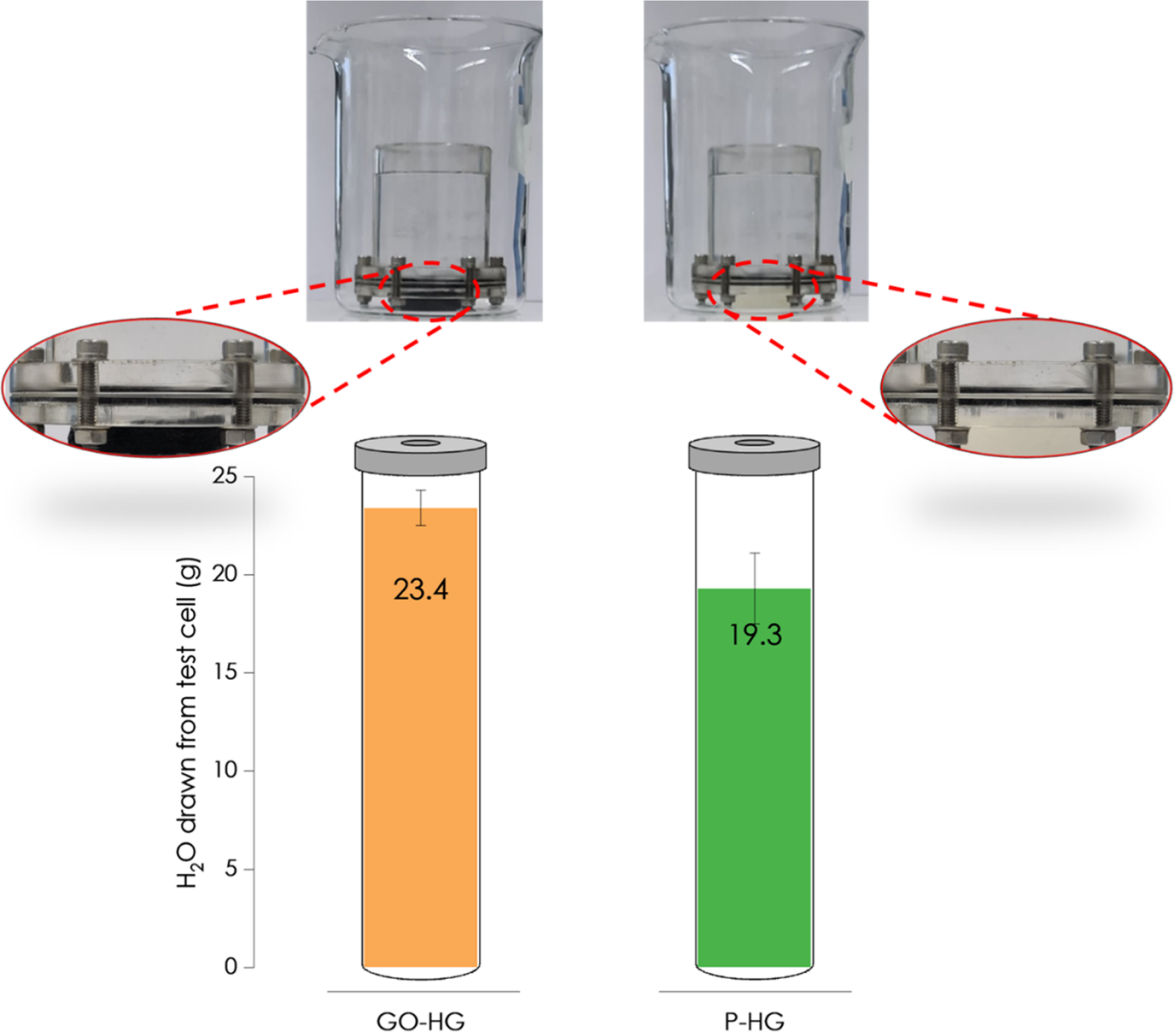
Water production results for the GO-HG and the P-HG. Error bars represent standard deviation.

Water production results are shown in Figure 11. Water production was quantitatively determined as the amount of water drawn by the hydrogel through an osmotic membrane over a 20 h period. The results from these tests showed the mean water production to be 23.4 ± 0.9 g and for the GO-HG hydrogel and 19.3 ± 1.8 g for the P-HG. Despite the reported higher hydrophilic nature of the P-HG hydrogel, the amount of water produced by the GO-HG is significantly more than that produced by the P-HG. This observation is attributed to the incorporated flexible GO nanosheets, which provided hydrophilic functional sites and increased the interconnectivity^75^ within the alginate hydrogel framework, resulting in the better water production performance of the GO-HG. The interconnected GO networks provided additional channels and paths for water to flow unhindered through the hydrogel, thereby increasing the amount of water transported by the GO-HG. The GO nanosheets are flexible and can partake in the formation of a polymeric network and easily achieve uniform distribution across the entire hydrogel. Different from most reported hydrogels that require external stimuli to undergo reversible volume change, water can be drawn through the membrane by the GO-HG and the P-HG in a continuous mode. No external stimulus is needed, and a hydrogel regeneration step is not required as the water flows through the interconnected water channels under gravitational force. It should be noted that the drawn water was not held within the hydrogels but was rather transported through the hydrogels and into the beaker for collection. The hydrogels are thus akin to conduits for transporting water. Therefore, there is no need for any dewatering step to recover the water drawn through the osmotic desalination membrane. This presents an advantage of using these hydrogels to draw water through osmotic membranes.

### Mechanism Analysis and Discussion

Osmotic pressure, which is a manifestation of chemical potential, is a primary driving force for water transport in osmotic membranes. The concentration difference of solutions at the osmotic membrane/draw agent interface behaves as a negative hydraulic pressure in osmotic membranes. Therefore, the possible driving force for water transport in the osmotic membranes can be considered as a hydraulic pressure gradient.^76^

Considering the test cell setup (Figure 5), in which an osmotic membrane is placed between the DI H_2_O and the hydrogel, a negative hydraulic pressure is responsible for the migration of water molecules through the osmotic membrane. Because there is no applied pressure on the DI H_2_O in the test cell, a gradient of hydraulic pressure in the osmotic membrane^76^ toward the hydrogel is developed by the induced negative pressure at the interface with the hydrogel. Water moves from the DI H_2_O side of the membrane to the hydrogel side of the membrane under this pressure gradient.

Recently, Song et al.^76^ postulated eq 2 to represent the water flux across an osmotic membrane.
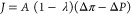
2where *J* is the water flux, *A* is the water permeability coefficient of the membrane, λ is the fraction of the membrane area that is unavailable for water flow because of cavitation, Δπ is the osmotic pressure difference across the membrane, and Δ*P* is the hydraulic pressure difference across the membrane.

In the osmotic membrane and hydrogel system, there is no pressure initially applied on the DI H_2_O side. Therefore, the Δ*P* term becomes null and eq 2 becomes
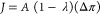
3

When the alginate hydrogels (P-HG and GO-HG) contact the osmotic membrane, they remove the liquid water from the membrane pores efficiently because of their strong hydrophilic properties. The membrane is regarded as the less hydrophilic region with relatively lower surface energy, whereas the hydrogel is regarded as the more hydrophilic region with relatively higher surface energy. Thus, a gradient in surface energy ensues. This surface energy gradient provides the driving force^77^ (eq 4) to transport water molecules from the surface of the membrane to the hydrogel.
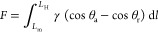
4where *F* stands for the driving force of water from the top of the hydrogel to the bottom of the hydrogel, γ stands for the surface tension of water, θ_a_ stands for the advancing contact angle of water droplets, θ_r_ stands for the receding contact angles of water droplets, and d*l* stands for the integrating variable along the length of the hydrogel, from the less hydrophilic membrane surface (*L*_m_) to the hydrophilic regions of the hydrogel (extending to the base of the hydrogel) (*L*_H_). It is worth noting that due to the permanent hydrophilic property and stable structure of the hydrogels, this equivalent hydraulic driving force, *F,* is maintained throughout the entire period of the experiments.

Additionally, the weight of the test cell over the hydrogel provides a gravitational force to expel the water from the hydrogel system. Consequently, the water drawn across the membrane will not be stored inside the hydrogel, but rather the water will flow freely through the interconnected water channels, allowing the continuous drawing of water without interruption. The presence of GO in the hydrogel improves the interconnectivity of the hydrophilic chains and makes the GO-HG more water-permeable than the P-HG, therefore enhancing the passage of water through the GO-HG. The abovementioned water production results suggest that the GO-HG and the P-HG can continuously draw water through a highly selective flat sheet cellulose triacetate (CTA) osmotic FO membrane owing to their permanent hydrophilic property and their stable structure. This shows the superiority of hydrogels over water-drawing solutions in maintaining the pressure gradient without being diluted by the permeated water. The hydrogel and selective osmotic membrane system can be used for water purification and desalination purposes.

## Conclusions

A pure calcium alginate hydrogel (P-HG) and a GO-incorporated calcium alginate hydrogel (GO-HG) were synthesized to draw water across a selective osmotic membrane. Morphological characterizations, carried out by SEM and AFM, displayed rough morphologies for both samples. Furthermore, AFM characterizations showed that the GO-HG had a higher surface roughness than the P-HG.

Water was successfully drawn through a selective flat sheet CTA osmotic desalination membrane by the hydrogels in a test cell setup as a result of the negative pressure induced by the surface energy gradient associated with the more hydrophilic hydrogel and the less hydrophilic membrane surface. It was found that the GO-HG was capable of drawing a 21.2% more water than the P-HG owing to the presence of flexible interconnected GO nanosheets with additional hydrophilic channels for water transport in the GO-HG. The mean amount of water drawn through the osmotic membrane by the GO-HG was 23.4 ± 0.9 g whereas that by the P-HG was 19.3 ± 1.8 g. The hydrogel and membrane system reported in this work exhibited the capacity to continuously draw water through a selective flat sheet CTA FO osmotic desalination membrane owing to the hydrophilic surface property of the hydrogels and the GO nanosheet-enhanced hydrophilic chains structure. The GO-enhanced hydrogel combined with the membrane has the potential as an alternative solution for water purification and desalination without applying external pressure.

## Supporting Information Available

Water contact angle results for P-HG and GO-HG hydrogels and water uptake results for P-HG and GO-HG hydrogels (PDF)
(PDF)
