# Supplementary material for: Graphene Oxide-alginate Hydrogel for Drawing Water through an Osmotic Membrane
Source: ACS Omega. 2022 Oct 18;7(43):38337–46. doi: 10.1021/acsomega.2c03138 (PMC9631913; doi:10.1021/acsomega.2c03138)
Supplement: Supplementary file 1 — ao2c03138_si_001.pdf [file ao2c03138_si_001.pdf]

Electronic Supplementary Information

Graphene Oxide-Alginate Hydrogel for Drawing Water Through an Osmotic Membrane

Adetunji Alabi[1], Cyril Aubry[2], Linda Zou[1]*

[1]Department of Civil Infrastructure and Environmental Engineering, Khalifa University of Science and Technology, PO Box 127788, Abu Dhabi, United Arab Emirates

[2]Department of Research Laboratories Operations, Khalifa University of Science and Technology, P.O. Box 127788, Abu Dhabi, United Arab Emirates

*Corresponding author

Email: linda.zou@ku.ac.ae

Water Contact Angle Measurements for P-HG and GO-HG Hydrogels

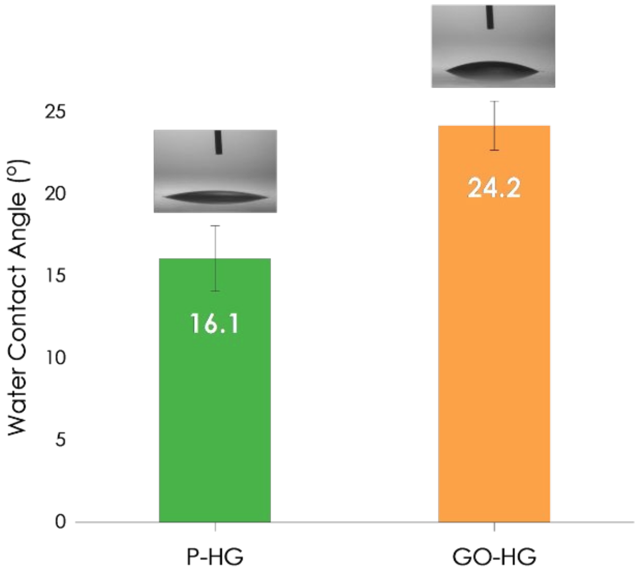

Figure S1 Water contact angle results for P-HG and GO-HG hydrogels. Error bars represent standard deviation.

Water Uptake Results

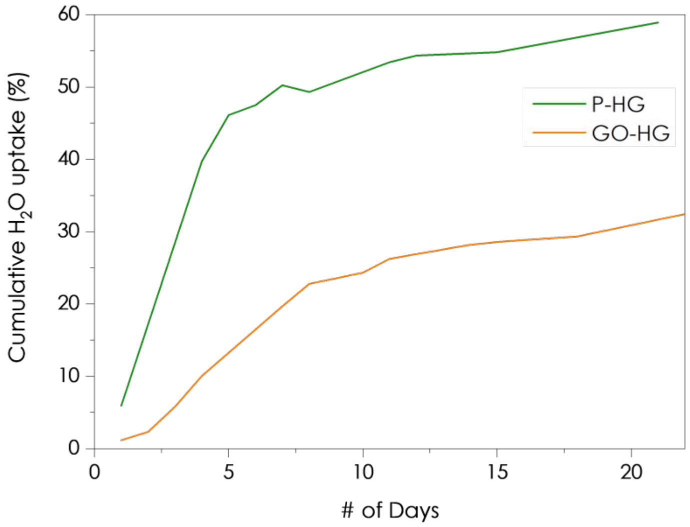

Figure S2 Water uptake results for P-HG and GO-HG hydrogels.
