# Supplementary figures and images for: Graphene Oxide-alginate Hydrogel for Drawing Water through an Osmotic Membrane
Source: ACS Omega. 2022 Oct 18;7(43):38337–46. doi: 10.1021/acsomega.2c03138 (PMC9631913; doi:10.1021/acsomega.2c03138)

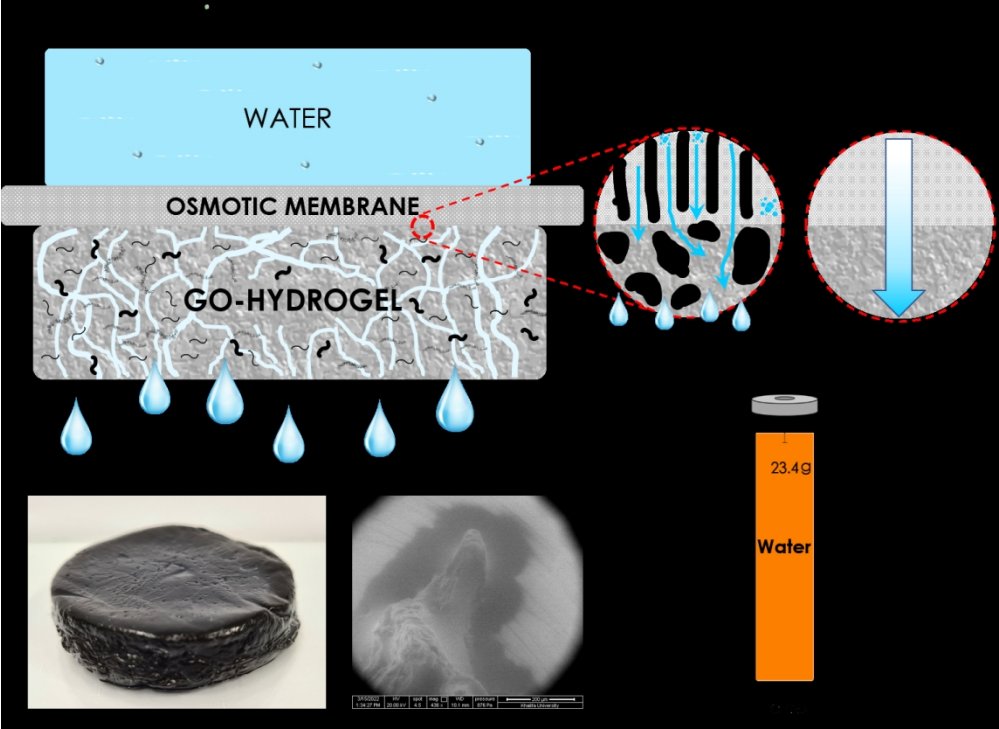

258x188mm (150 x 150 DPI)

Supplement: Supplementary file 2 — ao2c03138_si_002.pdf [file ao2c03138_si_002.pdf]
